# Nutrient supply and adequacy of macro- and micronutrients from Swedish agricultural production in 2024

**DOI:** 10.3389/fnut.2026.1796463

**Published:** 2026-03-30

**Authors:** Hedda Malmquist, Serina Ahlgren, Josefin Edwall Löfvenborg, Galia Zamaratskaia

**Affiliations:** 1 Department of Molecular Sciences, Swedish University of Agricultural Sciences, Uppsala, Sweden; 2 Division for Food and Drinking Water Supply, Swedish Food Agency, Uppsala, Sweden; 3 Division for Risk and Benefit Assessment, Swedish Food Agency, Uppsala, Sweden

**Keywords:** crisis preparedness, domestic food production, nutrient availability, nutritional security, nutritional self-sufficiency

## Abstract

**Introduction:**

Ensuring adequate nutrient availability from domestic food production is an important component of national food system resilience, particularly under scenarios of trade disruption or reduced imports. This study estimated the theoretical per capita nutrient availability from domestic Swedish food production under current peacetime conditions and compared it with population nutrient reference values.

**Methods:**

Calculations were based on official agricultural statistics, conversion factors for edible yields, assumptions on food losses, and food composition data. The analysis assumed current production volumes and an undisturbed food system to estimate the theoretical nutrient supply available from domestic production.

**Results:**

Sweden's peacetime food production has substantial capacity to meet or exceed population requirements for several essential nutrients. In particular, the estimated supplies of protein, carbohydrates, and dietary fiber comfortably exceeded recommended levels. Potential insufficiencies were identified for total fat, vitamin C, vitamin D, iodine, potassium, sodium, and selenium when considering domestic production alone and excluding imported or fortified products.

**Conclusion:**

These findings indicate that Sweden's domestic food production could support population nutrient needs for many essential nutrients but may not fully cover some vitamins, minerals, and fats. Maintaining nutritional security during trade disruptions may require additional food sources, including edible animal by-products and wild foods, or reliance on imports and fortification strategies.

## 1 Introduction

The adequacy of domestic food production for meeting national nutritional needs is fundamental for nutritional security, particularly as potential shocks such as natural disasters and pandemics present considerable risks to the resilience of global food systems (1). Recent global disruptions, including the COVID-19 pandemic and geopolitical conflicts, have revived debates about food self-sufficiency, with over a third of all countries unable to meet self-sufficiency for more than two of seven essential food groups (2).

Several studies have identified widespread nutrient insufficiencies. Passarelli et al. (3) examined the nutritional adequacy of national food supplies across 190 countries. It was found that many countries lacked sufficient levels of key nutrients, including vitamin D, vitamin E, calcium, and choline. That study used food balance sheet data and modeled nutrient availability against recommended intake levels. Surprisingly, even countries with high food availability often failed to meet micronutrient needs. Passarelli et al. (3) emphasized the importance of diversifying food production and improving access to nutrient-rich foods to enhance nutritional resilience.

The agricultural sector of Sweden plays an essential role in ensuring the nation's nutritional security, particularly during times of crisis. The adequacy of nutrients, derived from domestic agricultural production, is crucial for maintaining public health and resilience. In 2022, Sweden established a preparedness system aimed at enhancing the nation's ability to withstand threats and risks, prevent vulnerabilities, manage peacetime crises, and perform essential tasks during heightened preparedness. One of the designated preparedness sectors is “Food Supply and Drinking Water,” wherein agencies such as the Swedish Food Agency, County Administrative Boards, the Swedish Environmental Protection Agency, the Swedish Board of Agriculture, and the Swedish National Veterinary Institute collaborate to ensure the availability of safe food and water for the population during crises and periods of heightened preparedness (4).

The resilience of Sweden's food system depends on agricultural practices, climate, and policies, as well as efficient storage, processing, and distribution infrastructure (5–7). Key outputs include cereals, oilseeds, pulses, sugar beets, potatoes, vegetables, eggs, dairy, and meat, with cereals supplying food, feed, and biofuel production (8).

According to the Nordic Nutrition Recommendations (NNR) 2023 (9), adult macronutrient intakes should be balanced: 45–60 E% carbohydrates (≥25–35 g/day fiber), 25–40 E% total fat (< 10 E% saturated, 10–20 E% monounsaturated, 5–10 E% polyunsaturated), and 10–20 E% protein. The average requirement (AR) for protein is 0.66 g/kg/day, while the recommended intake (RI) covers 97.5% of the population. Reference values for essential vitamins and minerals are also provided to prevent deficiencies. Disruptions to food supply can increase the risk of inadequate nutrient intake, particularly for micronutrients, as observed during recent emergencies (10, 11). Evaluating nutrient availability from Swedish agriculture is therefore critical for public health planning (9).

The aim of the present study was to quantitatively assess whether Swedish food production in 2024 could meet the nutritional requirements of the Swedish population, including macronutrients (carbohydrates, fats, and proteins) as well as selected vitamins and minerals. 2024 was a normal agricultural year but still 5% below the 5-year average harvest of cereals. This study estimated the nutritional availability from Swedish food production and compared it with nutrient reference values. It is important to note that the calculations are based on peace-time conditions. In a crisis or war situation, trade disruptions may affect access to agricultural inputs and lower production substantially. However, peacetime production gives an indication of the nation's capabilities to produce food to it's population and can aid in preparedness planning. On other words, this study aims to contribute to nutritional preparedness planning by evaluating the capacity of Swedish primary production to supply the population with food.

## 2 Material and methods

This study estimated the nutritional availability from Swedish food production and compared it with nutrient reference values. Calculations on food production were based on official statistics on agricultural production, conversion factors for edible yields, food loss assumptions, and nutritional composition of food items.

### 2.1 Nutrient reference values

Calculations of the population requirements of carbohydrates and fats were based on the midpoints of the recommended intake ranges provided in the NNR 2023 (9), i.e., 52.5 E% for carbohydrates including 30 g dietary fibers, and 32.5 E% total fats of which less than 10 E% saturated fats, 15 E% monounsaturated fats, and 7.5 E% polyunsaturated fats. The energy requirement used in the calculations was 2,450 kcal per day, which is the assumed population average requirement in times of crisis with elevated physical activity demands (12). Energy requirements vary among individuals based on age, sex, body weight, height, physical activity, and environmental temperature. In normal peacetime circumstances, the requirement is about 2,350 kcal per day per capita. This number is based on a moderate physical activity level except for elderly aged 75 years and older for which a low level of physical activity was assumed. In a state of heightened alert, this average is estimated to increase by about 100 kcal due to increased physical activity, i.e., an energy requirement of 2,450 kcal per person per day. This figure is an average for the entire population, i.e., all age groups, based on the population structure in year 2020. The estimation also includes a proportion (based on national statistics) of pregnant and lactating women, which are groups with increased energy demands. During high alert, adults working in critical societal functions will need to be more physically active. On the other hand, others may become less active than in everyday life. The extra 100 kcal on average is based on the assumption that 750,000 people aged 18–60 years will be very physically active and the rest unchanged (12). To investigate the impact of higher energy requirements during a crisis, we also performed a sensitivity analysis using an elevated average energy intake of 2,700 kcal/day. This accounts for increased physical activity for a larger proportion of the population under extreme conditions, such as reliance on manual labor or limited mechanized transport. Under the 2,700 kcal/day scenario, the resulting reference intakes were approximately 50 g/day for protein, 354 g/day for carbohydrates, and 98 g/day for total fat, based on a protein requirement of 0.66 g/kg/day for a 76 kg individual and the same macronutrient energy distribution as in the baseline scenario.

Average protein requirements (0.66 g/kg/day) (9) were calculated for an individual weighing 76 kg, representing the average between Swedish men (84 kg) and Swedish women (68 kg) (13). Calculations for carbohydrates and fats were based on their energy contribution of 4 kcal/g and 9 kcal/g, respectively. The estimated average requirements of carbohydrates, fats, and proteins in grams per day are shown in Table 1.

**Table 1:** Recommended daily intake and estimated supply of nutrients from Swedish food production in 2024.

**Nutrient**	Recommended intake daily (g or mg^*^)	**Total production**	**Produced per person per day (g or mg)**
*Macronutrients and dietary fiber*			
Protein, g	50	3.30 × 10^11^ g	85.40
Total fat, g	88	2.63 × 10^11^ g	68.14
Saturated fat, g	< 27	7.74 × 10^10^ g	20.03
MUFA, g	41	1.08 × 10^11^	28.00
PUFA, g	20	5.32 × 10^10^ g	13.76
Carbohydrates, g	320	1.51 × 10^12^ g	390.11
Dietary fiber, g	30	1.48 × 10^11^ g	38.25
*Vitamins*			
Thiamin (B1), mg	1.2	6.61 × 10^9^ mg	1.71
Riboflavin (B2), mg	1.3	7.44 × 10^9^ mg	1.93
Vitamin B6, mg	1.6	6.72 × 10^9^ mg	1.74
Folate (B9), μg	300	1.46 × 10^12^ μg	378.05
Vitamin B12, μg	2.4	2.89 × 10^10^ μg	7.48
Vitamin A, RE	800	6.37 × 10^12^ RE	1,647.64
Vitamin C, mg	75	2.36 × 10^11^ mg	60.96
Vitamin D, μg	10	1.43 × 10^10^ μg	3.71
Vitamin K, μg	55	3.77 × 10^11^ μg	97.52
*Minerals*			
Iron, mg	9	5.25 × 10^10^ mg	13.59
Zinc, mg	9	4.91 × 10^10^ mg	12.70
Selenium, μg	55	2.08 × 10^11^ μg	53.90
Sodium, mg	1,500	1.76 × 10^12^ mg	456.39
Magnesium, mg	250	1.39 × 10^12^ mg	360.89
Potassium, mg	2,800	1.06 × 10^13^ mg	2,753.75
Calcium, mg	800	3.24 × 10^12^ mg	837.98
Phosphorus, mg	700	6.95 × 10^12^ mg	1,799.38
Iodine, μg	150	2.88 × 10^11^ μg	74.62

^*^Units as adapted from the recommended intake source. Recommended intake values and production data are derived from Nordic Nutrition Recommendations 2023 and Swedish production statistics 2024, respectively.

The estimated population requirement of vitamins and minerals was based on average requirements presented in NNR 2023 (9) and calculated as an average of the reference values for women and men in the age group 25–50 years (Table 1).

### 2.2 Data sources for agricultural production

Production statistics were obtained from the Swedish Board of Agriculture (14) and food composition data from the Swedish Food Agency (15). Production data for animal and plant-based products were primarily from 2024, with the exception of game meat, fish, and vegetables, for which 2023 data were used. A hypothetical yield reduction scenarios (20%−40%) were considered to examine how potential disruptions to domestic food production would affect nutrient availability. This sensitivity analysis allowed assessment of how baseline sufficiency estimates might change under more realistic crisis conditions.

### 2.3 Conversion factors

Recalculations of production data were conducted to estimate amounts available for human consumption. For meat, edible yields from carcass weight were assumed to be 70% for cattle, 86% for lamb, 68% boneless meat from pigs, and 15.4% edible by-products (16). For cereals, all exports were first subtracted, and the remaining cereal was then allocated to human consumption-−23% of wheat, 97% of rye, 20% of barley, and 28% of oats (other cereal uses are e.g., feed and biofuel). Export volumes were then added back under the assumption that they could be directed to human consumption. A 1:1 conversion factor between grain and flour was applied, as residues (husk, bran, etc.) were accounted for under food loss. Additional assumptions included a 40% yield oil from of rapeseed, 17% yield of sugar from sugar beet, and a starch yield of 25% from potatoes (17).

### 2.4 Food loss assumptions

Food loss factors were applied across the food chain up to the consumer level. For handling, storage, industry, and retail, loss rates were assumed to be 17% for cereals (including losses related to husk, bran, etc.), 7% for pulses, and 17% for fresh vegetables. At the retail level, losses were assumed to be 2% for vegetable oil and sugar, 9% for meat, 9% for fish, and 1% for eggs (17).

### 2.5 Nutrient composition and population reference

For each major food category, we combined nutrient composition values (carbohydrates, proteins, fats, vitamins, minerals) with the respective annual production data. The resulting figures represent the amounts of each nutrient available for the Swedish population of 10,587,710 in 2024 (18).

## 3 Results

Swedish domestic food production in 2024 comprised a diverse range of animal- and plant-based products, covering the major food groups present in the national supply (Table 2). The average quantity available for consumption per week and person varied across food types, with dairy and cereals accounting for the largest quantities, and products such as lamb, fish, and legumes representing lower levels.

**Table 2:** Swedish production of animal and plant foods for human consumption in 2024.

**Food category**	**Annual production, kg**	**Availability for consumption, kg/week/person**
Beef	97,664,000	0.177
Pork	204,960,907	0.372
Lamb	3,589,500	0.007
Poultry	140,240,100	0.255
Other meat	16,030,000	0.029
Eggs	114,015,600	0.207
Dairy	1,141,270,000	2.073
Fish	29,807,500	0.054
Cereals for human consumption	1,123,177,000	2.040
Cereals for export	519,700,000	0.944
Rapeseed oil	112,840,000	0.205
Sugar from sugar beets	360,230,000	0.654
Table potatoes	476,500,000	0.865
Potato starch	75,750,000	0.138
Peas and beans	74,536,000	0.135
Vegetables, berries, and fruit	391,686,000	0.711

**Figure 1:**
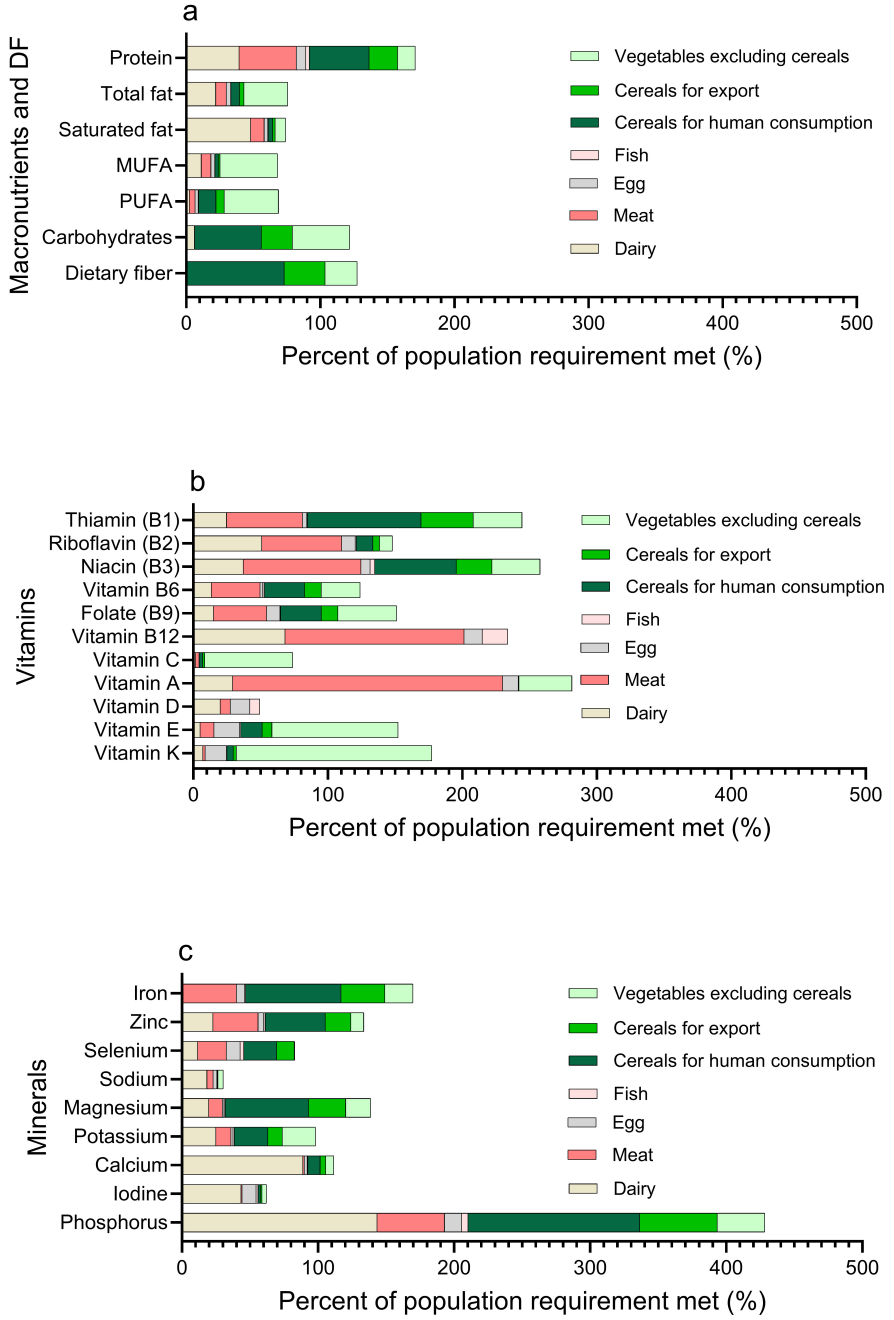
Nutritional adequacy of Swedish domestic food production under crisis conditions: **(a)** macronutrients, **(b)** vitamins, and **(c)** minerals compared to population requirements.

Table 1 and Figure 1a show macronutrient coverage from Swedish 2024 production vs. 2,450 kcal/day requirements: protein 171%, carbohydrates 122%, dietary fiber 127%, total fat 77%. Fat calculations used NNR 2023 midpoint (32.5 E% of 2,450 kcal). Under 2,700 kcal/day scenario, requirements increased to 354 g carbohydrates and 98 g total fat; coverage became 110% carbohydrates, 69% total fat (protein and fiber unchanged).

Protein supply was primarily fulfilled by meat, dairy, and cereals for human consumption. Dairy also contributed largely to total and saturated fat content (Figure 1a). Carbohydrates and dietary fiber reached the highest coverage via cereals for human consumption and vegetables, excluding cereals. Polyunsaturated (PUFA) and monounsaturated fats (MUFA) have the largest contributions from plant- and some from animal-based food.

In addition to macronutrients, the production of essential vitamins and minerals from Swedish domestic food sources revealed a mixed picture of nutritional adequacy (Table 1; Figure 1b). Several vitamins, including thiamin, riboflavin, niacin, vitamin B6, folate, vitamin B12, vitamin A, vitamin E, and vitamin K, were produced in quantities that exceeded or met the population's average requirements. Meat dominated the supply of vitamins A and B12, while cereals primarily contribute riboflavin and thiamine (Figure 1b). Vegetables, excluding cereals, contributed to vitamin K and vitamin E supply. However, notable insufficiencies were observed for vitamin C and vitamin D, with vitamin D being produced almost exclusively by animal-based foods and reflecting the lowest coverage among vitamins.

Assuming 20% reduction across all food groups (Table 2 production × 0.8), nutrient availability decreased proportionally, reducing coverage to 98%−137% for macronutrients and widening micronutrient gaps (vitamin D 78%, iodine 68%). A 40% reduction (production × 0.6) yielded 73%−103% macronutrient coverage with critical shortfalls (vitamin D 47%, iodine 41%). Exact values depend on differential impacts across food categories.

Mineral production adequately met or exceeded needs for phosphorus, iron, calcium, magnesium, and zinc but showed insufficiency for iodine, potassium, sodium, and selenium (Table 1; Figure 1c). Phosphorus largely originated from cereals and dairy, iron mainly from cereals and meat, and potassium and magnesium from vegetables. Sodium and iodine contributions were comparatively lower across food categories.

## 4 Discussion

### 4.1 Nutritional adequacy of Swedish domestic food production

The present analysis revealed that Swedish domestic food production in 2024 had substantial capacity to meet population nutritional needs under crisis conditions for most macronutrients. It also met the estimated population requirements for several essential micronutrients. Protein, carbohydrate, and dietary fiber supplies comfortably surpassed the estimated requirements, which is consistent with global patterns where high-income countries generally produce adequate amounts of these nutrients (1). However, insufficiencies were identified for total fat, vitamin C, vitamin D, iodine, potassium, sodium, and selenium. These gaps reveal challenges in the domestic food system. As a result, it may struggle to ensure full nutritional security during trade disruptions. Further, our study is based on present, undisturbed agricultural production. During a crisis, there may be events that lower production, for example, lack of inputs to agriculture, transport, electricity and IT disruptions. The capability to supply food to the population in crisis may in other words be lower than envisaged in this study. On the other hand, during a crisis other sources of food may be utilized for example edible animal intestines that today are used for biogas production could instead be eaten.

### 4.2 Vitamins D and A, and selenium

The insufficient domestic production of vitamin D observed in this study is particularly concerning for Nordic populations. Vitamin D is synthesized in the skin upon exposure to ultraviolet B radiation, but the Nordic countries are situated at latitudes where sunlight is insufficient for vitamin D production during winter months (19). Therefore, Sweden has a mandatory vitamin D fortification program (1 μg/100 g) for in low-fat milk (below 3% fat), fermented dairy, and margarines (20). Hence, the vitamin D content of dairy in this study partly originates from fortification and, consequently, disruptions in possibilities to fortify dairy products would lead to even lower vitamin D availability. Dairy is the dominant vitamin D source, and fortification significantly contributes to the calculated 14.3 billion μg supply (Table 1). In cases of limited fortification possibilities, vitamin D intake would primarily rely on natural sources such as fatty and lean fish, eggs and meat, worsening the existing 34% coverage shortfall. In contrast, vitamin A supply substantially exceeds requirements (189% coverage) primarily from meat. Dairy fortification contributes only a minor portion to this adequate supply, so its absence would have a limited impact. Vitamin D fortification policies vary among Nordic countries, ranging from mandatory widespread fortification to voluntary or minimal programs, which significantly affects population's vitamin D status (20). Vitamin D deficiency occurs in less than 20% of the population in Northern Europe, significantly lower than the 30%−60% prevalence in Western, Southern and Eastern Europe (21). The reliance on animal-based foods for vitamin D production identified in our results aligns with established dietary patterns in Nordic countries, where fatty fish, egg yolk, and fortified dairy products serve as primary vitamin D sources (9). The supply of vitamin D in Sweden could increase if, for example, marine production increases. There is potential for increased production of fish (marine and aquaculture) and marine products (seafood, algae, etc.), as well as increased use of side streams from fish processing for human consumption, without threatening sustainability (22).

Selenium is found in almost all foods, but the levels vary. In Sweden, the soil is low in selenium, and vegetables grown in Sweden therefore have low levels. The foods that contain the most selenium are fish, shellfish, organ meats, nuts/seeds, lentils, eggs and cheese (23). Selenium insufficiency represents another critical vulnerability. Suboptimal selenium status is widespread throughout Europe, with selenium levels notably lower than those in the United States, and Eastern Europe having lower average selenium intake than Western Europe (24). Finland initially had the most insufficient selenium intake in Europe but addressed this through fortifying fertilizers with selenium, resulting in improved population selenium status (25). In Sweden, selenium intake is relatively low, which may increase the risk of deficiency (26–28).

### 4.3 Vitamin C

The inadequate production of vitamin C highlights dependency on imported fresh fruits and vegetables, categories where domestic production may be seasonally limited in Nordic climates. However, in our analysis only official agricultural data was included, wild berries were not included. Sweden is one of the largest producers of wild berries in Europe, mostly bilberries, lingonberries, crowberries, and cloudberries, and there is a large potential to increase the domestic production of vitamin C. Wild berry yields (fresh weight) in Sweden are very large, approximately 336 million kg of bilberry and 382 million kg of lingonberry annually (average 2015–2019) (29). With 8 mg/100 g vitamin C content (30), full utilization could theoretically supply 20% of the population vitamin C requirements (80 mg/day). Energy contribution remains negligible, covering below 0.4% of national energy needs.

Vitamin C adequacy has been defined as plasma concentrations greater than 50 μmol/L, yet disparities in vitamin C status exist globally, with dietary intakes and processing methods significantly affecting availability (31). Among immune-health nutrients, vitamin C inadequacy affects approximately 46% of adults in populations with insufficient fruit and vegetable consumption (32).

### 4.4 Iodine

The insufficient iodine production identified in this analysis reflects a broader European challenge. Mild to moderate iodine deficiency is widespread among adults in Europe, and Norway has not implemented mandatory iodine fortification policies, making populations following plant-based diets particularly vulnerable (33). Sweden has an iodized salt program, and overall iodine intake is generally adequate, though mild deficiency has been observed in some groups, including school-age children and pregnant women (34, 35). Analysis of nutrient intake inadequacy in Europe applying reference values according to NNR 2023 demonstrated that the mean prevalence of inadequacy exceeded 20% for iodine across multiple population groups (28). The primary dietary sources of iodine in Nordic countries are dairy products and seafood, making domestic production capacity in these categories critical for iodine sufficiency during crisis scenarios.

### 4.5 Sodium

Sodium intake in Sweden predominantly originates from processed foods, which typically provide over two-thirds of total dietary sodium (36). However, Sweden imports approximately 500,000 metric tons of salt annually (37) with no significant domestic production due to geological limitations (no salt deposits). This creates a vulnerability in domestic nutritional security: for sodium to be present in processed foods, salt must be consistently available within Sweden's borders, and any disruption in salt imports could severely impact the national food supply. These risks highlight the importance of considering strategic salt reserves as part of nutritional security planning. Future research should more closely assess both the source of salt inputs and the potential for disruption, as well as the degree of food processing, to refine estimates of sodium availability under crisis or import interruption conditions.

### 4.6 Nutritional needs of vulnerable populations

The population-level analysis uses average requirements for a 76 kg adult, but vulnerable groups face distinct nutritional challenges during crisis rationing. Children require higher nutrient density per kg body weight, particularly protein, calcium, iron, and vitamins A and D for growth, exacerbating shortfalls in vitamin D, iodine, and selenium identified here (9). Older adults face unique nutritional challenges for muscle and bone health, including higher protein requirements for sarcopenia prevention (45). Pregnant and lactating women face heightened demands for folate, iron, iodine, and omega-3 during key developmental periods (9). Equitable per-capita food distribution would provide these groups proportionally less of their specific requirements from the same production pool, amplifying nutritional vulnerability beyond aggregate sufficiency estimates. Even in a welfare state like Sweden, dietary quality issues persist; nearly 40% of total energy intake among adolescents comes from low-nutrient foods and beverages, indicating suboptimal nutrient patterns in some population groups that could exacerbate vulnerability during crises (38). Such dietary patterns may increase the risk of inadequate micronutrient intake under crisis conditions, when food variety and availability are further constrained.

### 4.7 Food system resilience and crisis preparedness

The present findings underscore the importance of food system resilience in the context of increasing global uncertainties. Food systems are increasingly exposed to disruptions and shocks, with recent conflicts and pandemics increasing concerns about the ability to secure food availability at stable prices (39). A nation's nutritional self-sufficiency emerges as a vital determinant of food security (40), however most countries have trade of food e.g., in Sweden there is for obvious reasons no production of coffee, tea or cocoa. However, there is a large production of cereal that goes for export. This export could be used domestically, given that Swedish infrastructure (milling/processing) rapidly could pivot these volumes for human consumption during a crisis. Two facts points to this possibility; there is an overcapacity in the Swedish milling sector in the southern part of the country, and there are governmental plans to invest in mobile mills for preparedness reasons in the northern part of the country (41).

A resilient food system requires finding a balance between self-sufficiency and external trade. Trade also bring stability in food supply if harvests fail in one country, it may not do so in others. Key elements of food system resilience include system thinking through science and communication, redundancy of activities and networks, diversity of production and partners, and buffering strategies (39).

In a crisis preparedness perspective, it may, however, be good to aim for higher levels of self-sufficiency in agricultural production inputs to ensure the potential to maintain local production during prolonged crises (42).

### 4.8 Food system vulnerabilities and resilience considerations

While the present analysis provides a baseline estimate of nutrient availability based on current production volumes, it does not account for the vulnerability of critical agricultural inputs and infrastructure. National food systems are vulnerable not only due to production capacity but also due to interdependencies among inputs and infrastructure, a key theme in food system resilience research (43). Swedish food production depends on external inputs such as mineral fertilizers, fuel, imported feed components, plant protection products, and spare parts for machinery. In addition, food processing, storage, and distribution rely on stable electricity supply, transport systems, and digital infrastructure. Disruptions to these systems during a severe crisis could substantially reduce effective food availability, even if primary production capacity remains unchanged. Therefore, future resilience assessments should integrate both nutrient supply potential and system-level robustness.

### 4.9 Policy implications and future directions

The identified nutritional gaps suggest several policy interventions to enhance Sweden's nutritional security during crisis scenarios. Expanding domestic production of nutrient-dense foods, particularly those rich in fat, vitamin C, vitamin D, iodine, and selenium, should be prioritized. Interventions to improve nutrition security should consider biofortification, yield gap closure, and increased production of crops rich in inadequate nutrients while avoiding trade-offs with environmental sustainability (1). Biofortification of crops with selenium and iodine through agronomic strategies may represent a cost-effective approach to increasing daily intake of these essential trace elements (44). For vitamin D, policy options can include optimizing fortification programs or promoting increased production and consumption of vitamin D-rich fish, seafood, and algae products. Biofortification can be a way to address nutritional deficiencies, however it can be problematic if import is limited since fortification agents can be dependent of imports.

### 4.10 Limitations

This study has several limitations. The assessment is based on production data that may not capture all food sources, particularly from household gardens, wild production and minor crops. Therefore, the amount of nutrients might be higher because of higher food production than the calculations counted with. The study assumed even distribution of nutrients across the population, which may not reflect actual accessibility differences due to income, age, geography, or other socioeconomic factors. Additionally, the analysis does not account for the dependency on imported production inputs such as fertilizers, energy, and animal feed, which could significantly affect domestic production capacity during extended crisis scenarios. Furthermore, this study did not distinguish between processed and unprocessed foods, which is an important consideration as food processing can significantly affect nutrient content and bioavailability. Processing methods such as heating, drying, milling, and storage can lead to substantial losses of heat-sensitive vitamins (particularly vitamin C, thiamin, and folate) and may alter the bioavailability of minerals and other nutrients. When assessing the nutrient content of milk, fortified milk was used in the calculations. This milk is enriched with vitamin D and in some cases vitamin A, which can result in overestimation of the amounts of these vitamins supplied by domestic production. Therefore, the reported levels of vitamin A and D may be higher than what is naturally present in unfortified milk and should be interpreted with caution. It is important to emphasize that the calculations and nutrient availability discussed in this report are based on production data from 2024, which represented a normal agricultural year with generally favorable growing conditions. However, the supply of nutrients from Swedish agriculture and animal production fluctuates from year to year, depending on factors such as climate, weather, input availability, and land use. For a more robust and realistic assessment of Sweden's long-term food preparedness, calculations should ideally include data spanning multiple years or varying growing conditions, in order to enable meaningful comparisons and trend analyses.

## 5 Conclusion

Swedish domestic food production demonstrated considerable capacity to meet the population's nutritional needs for macronutrients and many micronutrients during 2024, during peace-time conditions when production inputs were available. This result suggests that, if Swedish production under crisis or war matches production levels seen in 2024, Sweden would have the capacity to cover the population's requirements in many nutrient categories, given that there is access to agricultural inputs such as diesel and fertilizers. However, even during peace-time critical insufficiencies in fat, vitamin C, vitamin D, iodine, selenium, and certain other nutrients highlight the need for strategic interventions to enhance nutritional resilience.

## Data Availability

Publicly available datasets were analyzed in this study. This data can be found here: https://jordbruksverket.se/om-jordbruksverket/jordbruksverkets-officiella-statistik/jordbruksverkets-statistikrapporter/statistik/2025-12-11-livsmedelskonsumtion-och-naringsinnehall.–uppgifter-till-och-med-2024.
